# pbcftools: parallel execution of bcftools for large variant call sets

**DOI:** 10.64898/2026.08.03.742604

**Published:** 2026-08-08

**Authors:** Ge Zhang

## Abstract

**Summary:**

bcftools is the standard toolkit for handling VCF and BCF variant files, but it processes records on a single core; its --threads option speeds up only compression of the output, not the work done on variant records. Processing large call sets is therefore slow, and users often divide the genome and reassemble the results by hand. We present pbcftools, a Perl wrapper that does this automatically: it splits the genome into chunks, runs an ordinary bcftools command on each in parallel, and reassembles the outputs by a method suited to the data type. Across Linux servers, Windows/WSL2 workstations and Apple laptops, with bcftools 1.21 to 1.24, parallel output was identical to serial output for every command tested. On 1000 Genomes Phase 3 data, operations writing compressed VCF ran 10.8 to 21.1 times faster with 32 cores and up to 35.4 times with 64, those writing text 3.7 to 12.8 times, and merging 100 VCF files 19.2 times. pbcftools also runs on LSF and Slurm clusters.

**Availability and implementation:**

pbcftools is written in Perl (>= 5.16) and requires bcftools; local parallel execution also requires Perl module Parallel::ForkManager. It is released under the MIT license at https://github.com/zhangge-uc/pbcftools (DOI: 10.5281/zenodo.21780361).

## Full Text Availability

The license terms selected by the author(s) for this preprint version do not permit archiving in PMC. The full text is available from the preprint server.

